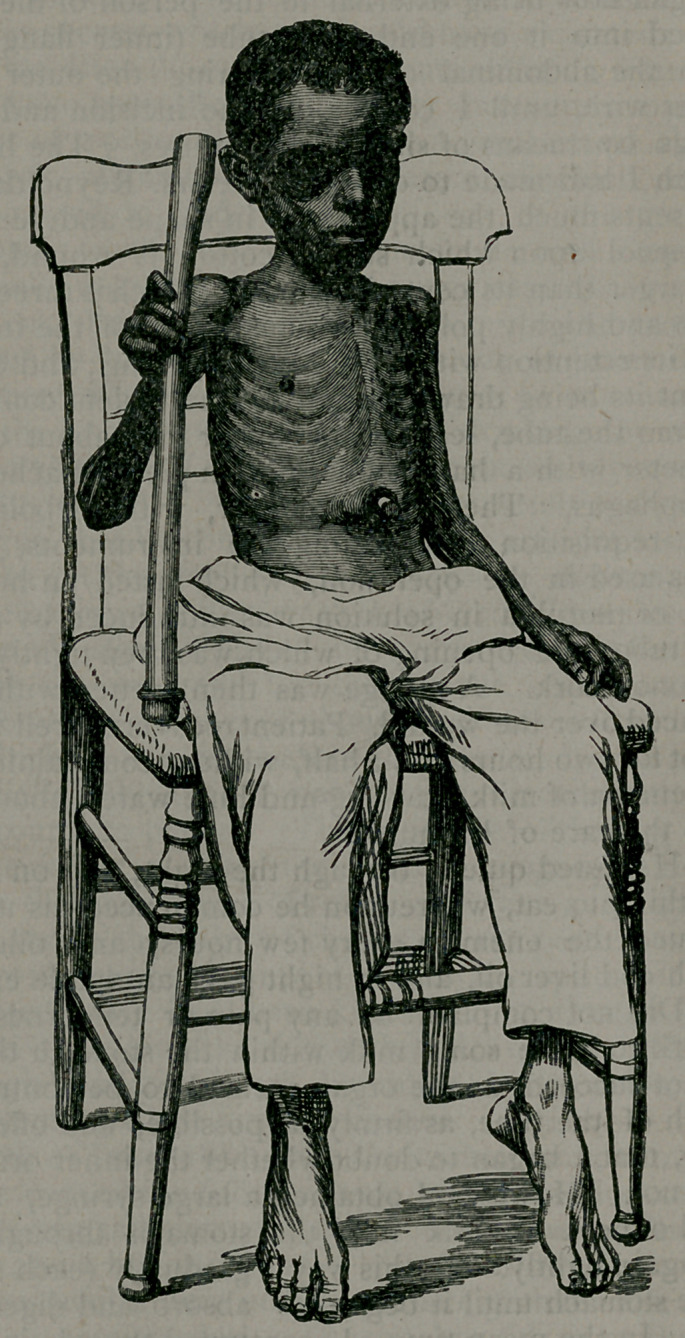# Gastrotomy or Gastrostomy

**Published:** 1881-01-20

**Authors:** L. L. Staton

**Affiliations:** N. C.


### GASTROTOMY OR GASTROSTOMY

BY L. L. STATON, M D., OF N. C.

I wish to place on record the particulars of a case which recently came under my care, considering it (as I do) to be the duty of every physician and surgeon to contribute his mite, however small and inglorious, to the relief of suffering humanity, and to advance the interest of the noble profession to which I have the honor to belong. The case to which I allude is unique in many respects, and of quite rare occurrence, and I find only a few cases mentioned in the standard authorities, and the medical periodicals of an extensive library to which I have had access through the courtesy of a medical friend, and notably among these few cases, is that of Dr. F. F. Maury, of Philadelphia, *American Journal Medical Science*, April, 1875, page 365, the first case of gastrotomy performed in this country for stricture of the œsophagus, the patient surviving the operation fourteen hours. I am quite aware that the cases have been few in which the operation ever proved a permanent benefit. The operation has been justified, but has never met with that success which we should have expected; and I fear that it has been too often the case that patients suffering from stricture of the œsophagus have been allowed gradually but surely to starve to death. The dangers of septicæmia are now to a great extent obviated by Lyster's antiseptic method, in consequence of which one is now very much encouraged to undertake operations of the gravest character. The question as to the value and benefits derived from the operation will be partially answered by the following case:

The patient, Lewis Lyon, colored, a boy eight years of age, was brought to me by his father on the first day of June, 1880, at which time the patient was almost dying of hunger on account of a cicatrized stricture of the œsophagus, the result of drinking, by mistake, a large quantity of a solution of commercial concentrated lye ( solution caustic soda), in August, 1879, which had so completely and gradually closed the œsophagus, that he could not then, June 1st, swallow anything, nor was I able to get the smallest bougie through the stricture. He was very much emaciated and so weak that he could not raise himself when down; but could stand if placed upon his feet. After exhausting all the means at my command for dilating the stricture or obstruction, which was found to commence about three inches from the gullet, (the extent of which it was impossible to ascertain), and failing to pass even the smallest bougie, I was fully convinced that gastrotomy was the proper course to pursue. The condition of the boy was such, that I gave to the father a very unfavorable prognosis, but advised an operation as the only means of relief, and that a barely possible one. The patient was in the habit of chewing every particle of food he could obtain, but without any attempt at swallowing, spitting it out as soon as well masticated. He had been kept alive for the last few months by enemata, and by rubbing the skin with cod liver oil. Here we have a case of aphagia rendering death imminent by inanition, and I determined to give my patient his only chance.

On the 17th of June, 1880, with the assistance of two of my medical friends, both concurring fully with me in the justification of the operation, after having administered chloroform, I proceeded to divide the skin for two and a half inches in a diagonal direction, from right to left, under the cartilaginous portion of the eighth left rib, and as near to the sternum as possible, but a finger's breadth from the median line. The walls of the abdomen being very thin, were divided in the same line without hæmorrhage. I did not follow Amusat's plan, or the operation advised by Sedillot in gastrotomy; but proceeded as here described (with the approval of the gentlemen present) as being the most feasible under the circumstances. I then carefully introduced two fingers to examine for the stomach, and coming in contact with a hard and seemingly solid mass, that felt more like a fibrous tumor than a stomach, I drew it through the opening in the abdominal walls, and found it to be the organ in question. It was firmly contracted, about two and a half inches in length, and about one and a half inches wide. With the view of making a permanent fistula, I made an incision about three-fourths of an inch long, parallel with the long diameter of the viscus, near the smaller curvature as advised by Prof. Verneuil, of Paris. The organ now being external to the person of the patient, I easily introduced into it one end of the tube (inner flange), and returned it within the abdominal cavity, securing the outer flange by means of a silver wire, until I could close the incision and make firm the surroundings by means of silver wire sutures. The hard rubber tube used, which I had made to order by Messrs. Reynolds & Co., of New York, presents much the appearance in shape and length of the small wooden spool upon which sewing cotton is wound, each end (flange) being larger than its central diameter, which is three-eighths of an inch, smooth and highly polished. The object of the inner flange being to insure its retention within the gastric opening; and of the outer flange to prevent its being drawn within by the violent contractions of the viscus. From the tube, leads a soft rubber pipe about one half of an inch in diameter with a hard rubber mouth-piece attached, making an artificial œsophagus. The steam atomizer, with carbolized water, was in constant requisition, disinfecting the instruments, my hands, and the sponges used in the operation, which lasted an hour. One-sixth of a grain of morphia in solution was introduced by a small syringe, into the tube, the opening of which was then tightly closed by means of a common cork. A sponge was then wetted with the disinfectant, and placed over the wound. Patient recovered well from anæsthesia, and slept for two hours and a half, without complaining of pain. I then gave an enema of milk, raw egg and lime water, about ℥ij., and then left him to the care of his nurse.

June 18th.—He rested quietly through the night, and on awakening called for something to eat, whereupon he commenced his usual chewing. I continued the enemata every few hours, and oiled the skin twice a day with cod liver oil, and at night gave an opiate enema.

June 19th.—Did not complain of any pain or tenderness. I then removed the cork to place some milk within the stomach through the tube; but did not succeed, as the organ seemed to be contracted over the inner mouth of the tube, as firmly as possible, and offered such a great resistance, that I began to doubt whether the inner orifice was in the stomach or not. However I obtained a large syringe, and forced about four fluid ounces of milk into the stomach through the tube, then corked it again tightly. In this way I gradually (each succeeding day) dilated the stomach until it began to absorb and digest the food placed therein. In the mean time, I continued the administration of nutritious enemata, such as milk, yolk of eggs, beef essence, etc., made as warm as could be tolerated, and oiled the surface of the body, freely with cod liver oil.

In a few days the patient began to show an appreciable increase of flesh, and a decided improvement of strength. However, he has not been able to digest the coarser foods, but is rapidly improving, and I

am now (August 18th), two months after the operation, feeding him upon substantial diet; first letting him chew it all, and then eject it nto the stomach through the rubber pipe, made by Reynolds & Co., of New York.

The boy has recovered very slowly from his enfeebled condition; has never had any peritonitis—a most fruitful source of death after this operation—or inflammation of any of the tissues, save an unhealthy granulation around the tube which I controlled by the nitrate of silver.

Mr. Thomas Smith (case of gastrotomy) points out that with one exception, every patient who has survived the first three days after the operation, had died of peritonitis. My patiént came very near dying from an over quantity of grated ham and biscuit, three weeks after the operation. His bowels for the first few weeks, moved about once a week, but he is now, at the date of this paper, having a gentle action.

once a day. I sent a photograph of the boy taken before the operation, showing his impoverished condition, and another, showing the operation and the artificial œsophagus.

How he is nourished will scarcely require explanation. In feeding, the "œsophagus" is simply removed to the outside of his person, for it is rubber, instead of being muscular tissue. The boy, after thoroughly masticating his food, simply spits it through the tube into the stomach in a semi-fluid state. In this manner his life has been saved, and he is now independent of the stricture of the œsophagus. The benefits to him of the operative procedure by the mechanical means devised, cannot be overestimated.

The practical result of my case has been, unquestionably, the prolongation of life, which is the great desideratum of the medical man, and none the less, the desire of the patient; but whether the life of the subject of this report is "worth living," will be a matter which will be more easily, and perhaps more readily determined by Lewis Lyon, than myself.

*Nous verrons.* I have done my part.—*N. C. Med. Journal.*

## Figures and Tables

**Figure f1:**
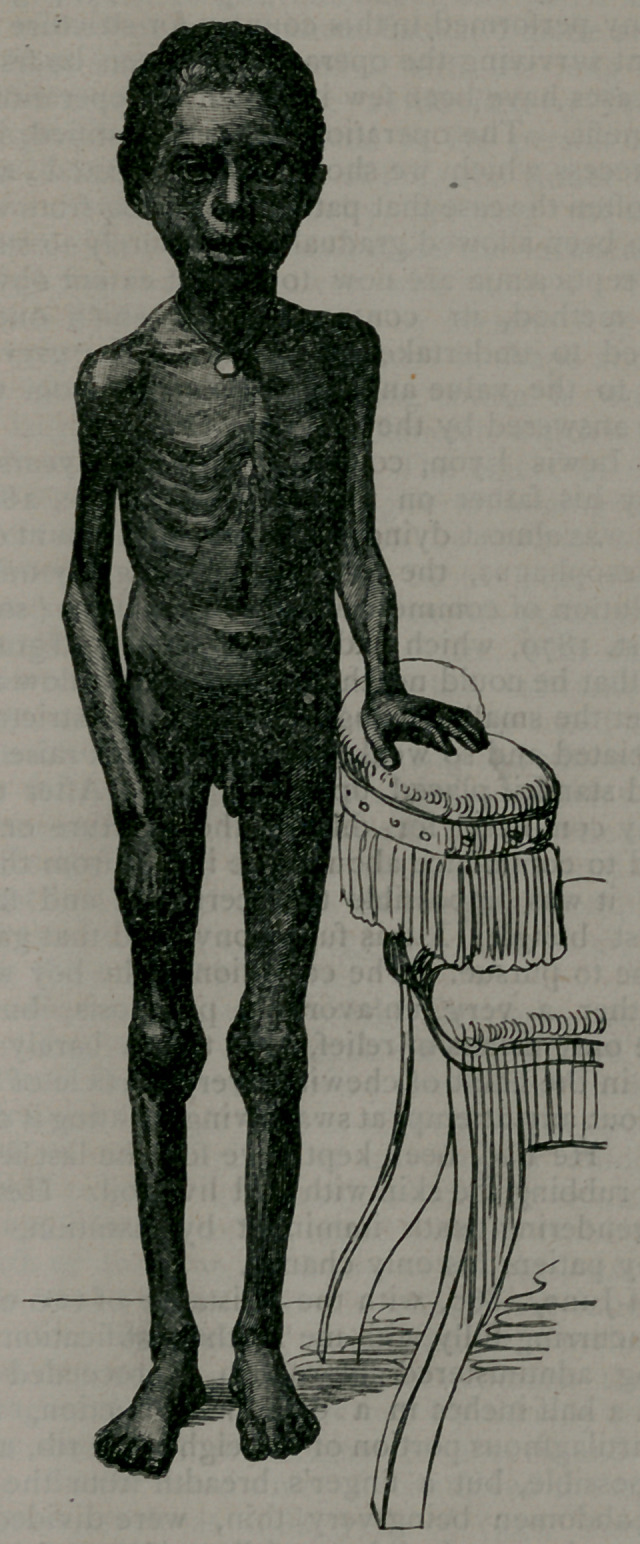


**Figure f2:**